# Direct nose to brain delivery of small molecules: critical analysis of data from a standardized *in vivo* screening model in rats

**DOI:** 10.1080/10717544.2020.1837291

**Published:** 2020-11-10

**Authors:** Deborah Dhuyvetter, Fetene Tekle, Maxim Nazarov, Rob J. Vreeken, Herman Borghys, Frederik Rombouts, Ilse Lenaerts, Astrid Bottelbergs

**Affiliations:** a Non Clinical Safety, Janssen R&D, Beerse, Belgium; b Non Clinical Statistics, Janssen R&D, Beerse, Belgium; c Open Analytics, Antwerp, Belgium; d Drug Metabolism & Pharmacokinetics, Janssen R&D, Beerse, Belgium; e Neuroscience Therapeutic Area, Janssen R&D, Beerse, Belgium

**Keywords:** Drug delivery, nose to brain, intranasal, rat, brain targeting, direct CNS transport

## Abstract

The blood–brain barrier (BBB) is often a limiting factor for getting drugs in the brain. Bypassing the BBB by intranasal (IN), or also called nose to brain (NTB), route is an interesting and frequently investigated concept for brain drug delivery. However, despite the body of evidence for IN drug delivery in literature over the last decades, reproducibility and interpretation of animal data remain challenging. The objective of this project was to assess the feasibility and value of a standardized IN screening model in rats for the evaluation of direct brain delivery. A chemically diverse set of commercial and internal small molecules were tested in the *in vivo* model with different doses and/or formulations. Data were analyzed using different ways of ratio calculations: blood concentration at time of sacrifice, total exposure in blood (area under the curve, AUC) and the brain or olfactory bulb concentrations. The IN route was compared to another parenteral route to decide if there is potential direct brain transport. The results show that blood and tissue concentrations and ratios are highly variable and not always reproducible. Potential direct brain delivery was concluded for some compounds, however, sometimes depending on the analysis: using blood levels at sacrifice or AUC could lead to different conclusions. We conclude that a screening model for the evaluation of direct brain transport of small molecules is very difficult to achieve and a conclusion based on a limited number of animals with this variability is questionable.

## 1. Introduction

The blood–brain barrier (BBB) is a highly regulated and an efficient barrier that provides a sanctuary to the brain. It is designed to regulate brain homeostasis and to permit highly selective transport of molecules that are essential for brain function (Sweeney et al., 2019). This implies several restrictions on the properties of drugs intended to treat central nervous system (CNS) based maladies. Several strategies are currently being investigated preclinically for drug targeting into the brain, but they often involve invasive techniques, such as direct injection into the brain (Guerin et al., 2004), infusion or global disruption of the BBB (Khan et al., 2017). Intranasal (IN) delivery could offer an opportunity to serve as a noninvasive direct route to the CNS since the nasal cavity is innervated by olfactory and trigeminal nerves creating pathways for transport (Balin et al., 1986; Minn et al., 2002; Hanson & Frey, 2008; Pires et al., 2009). This is a very attractive concept in drug development, having the advantages of avoiding the hepatic first-pass metabolism, potentially lowering the systemic exposure, circumventing the BBB and ease of self-administration. The IN route can also be used as alternative to intravenous delivery in specific cases such as emergency situations (Bailey et al., 2017). Over the past decades, hundreds of academic research papers have described studies on the nose to CNS route showing promising results in preclinical efficacy models and raising a lot of interest in the neuroscience field.

The exact mechanisms of IN drug delivery to the CNS are not entirely understood, but it has been accepted that drugs deposited on the nasal mucosa could reach the brain using two main transport routes: (a) systemic absorption from the nasal respiratory epithelium followed by transport across the BBB and/or (b) direct transport via peri-neuronal and peri-vascular channels associated with olfactory and trigeminal nerves, bypassing the BBB (Thorne et al., 1995; Hanson & Frey, 2008; Dhuria et al., 2010; Lochhead & Thorne, 2012; Crowe et al., 2018). The average amount of the nasal dose that reaches the brain after IN administration is reported to be small (below 1%) (Landis et al., 2012) limiting the applicability to extremely potent molecules. Also, various physicochemical properties of the molecule and physiological and anatomical factors potentially influence the drug transport and uptake (Ugwoke et al., 2005; Vyas et al. 2006; Pires et al., 2009; Lee et al., 2010).

Despite the entire field’s breakthroughs, showing IN delivery remains challenging, mainly due to reproducibility. The myriad of dosing forms (spray/catheter/pipet/instrumentation or specialized delivery systems) and diversity in sampled tissues (whole brain/brain areas/cerebrospinal fluid/brain extracellular fluid/blood/plasma) in conjunction with formulation properties might be the origin of non-reproducibility, since these details are not always available. Several reviews pointed out features necessary for a realistic experimental design to study nose to brain (NTB) drug transport (Merkus & van den Berg, 2007; Kozlovskaya et al., 2014). Taken together these evaluated more than 100 papers on experimental design, formulations, quantification methods, and data analysis. The findings suggest that there remains an unmet need for a reliable methodology and data interpretation of IN delivery to the brain but that this field does have the potential of delivering solutions for the treatment or diagnosis of CNS disorders.

Intrigued by the NTB potential, we decided to investigate this delivery approach to assess the feasibility and the value of developing such a platform for CNS small molecules. The goal would be to have a screening method to evaluate the potential benefit of dosing NTB. In this attempt, we evaluated the deposition site, dose volume, formulation, and sampling technique to optimize and standardize an NTB animal model. Subsequently, some commercial and internal compounds were tested using this model system. This paper discusses a proof of concept for IN direct brain transport in a rat model. Next to a standardized method for optimal NTB delivery, a critical view on the data is presented.

## 2. Materials and methods

### 2.1. *In vivo* studies

All studies were conducted in accordance with Directive 2010/63/EU of the European Parliament and of the Council of 22 September 2010 on the protection of animals used for scientific purposes, and with ‘the Appendices A and B’, made at Strasbourg on March 18 1986 (Belgian Act of October 18 1991). The protocol and study design were approved by the Janssen Ethical Committee for Animal Experiments.

*Animals*. The studies were performed in naïve male rats (Sprague Dawley, supplier Charles River France, Écully, France) with a body weight of 220–280 g (aged 6–8 weeks). After entering the facility and being health checked, the animals were allowed to acclimatize for at least five days. The animals were group housed (3–5 animals) in individually ventilated cages (IVCs) on a 12 h day–night cycle. Standard lab diet (Safe, type A05 SP-25) and tap water were available *ad libitum*. The choice of species was based on practical considerations, accessibility of the nasal cavity and the option for cerebrospinal fluid sampling (however not performed). Male rats are most frequently used in our facilities; hence, the gender choice was solely to optimize animal use.

*Study design*. The animals were dosed under isoflurane (ISOFLO^®^, Abbott, Chicago, IL) inhalation anesthesia (4% for induction, 2% for maintenance) by different routes: intravenously (IV), subcutaneously (SC), IN targeting the olfactory epithelium (= intranasal nose to brain: IN-NTB) or IN nose drop covering the respiratory and olfactory epithelium (IN-ND). The groups consisted of 3–5 (IV, SC) or 5–10 (IN) animals per end point. Dosing was done per cage. A summary of the total number of animals can be found in Supplementary data (1°). Group size was chosen based on the expected pharmacokinetic variability. Since the goal was to standardize this method as a screening for a fast brain delivery, early time points were chosen: blood samples were taken at 1, 3, and 5 min or at 1, 3, 5, 10, and 20 min after the dosing procedure. However, in one study later time points (30, 60, 180, and 360 min) were used on request for a specific compound. These data are included in the analysis. After the last blood sample (end points 5, 20 min and all later time points), the animals received an IV injection (tail vein) of heparin (1000 IU/kg, LEO^®^ 5 mL, 5000 I.E./mL) and were euthanized under isoflurane anesthesia by decapitation to collect the brain and olfactory bulbs. Heparin was used to maximize blood drainage from the brain since brain perfusion was not feasible in a screening platform.

*IV and SC dosing*. Animals were dosed in the saphenous vein (IV) or in the neck region (SC).

*IN-NTB dosing*. To ensure the most accurate targeting of the olfactory bulb, a method optimization was performed first. The animals were placed in supine position with the head at a 45° angle. A 1.2 French catheter (Instech Solomon PUFC-C20-10, funnel Cath tapered PU cath 2 F narrowing to tip of 1.2 F) was placed at different depths (ranging from 2 to 2.5 cm) in the nostril to target the olfactory epithelium. Different volumes (ranging from 15 to 50 µL per nostril) of methylene blue solutions were then infused over a period of 60 s (by constant rate infusion pump, with rate depending on volume to be infused) in each nostril consecutively (first right then left nostril). Evaluation was done based on visual inspection (blue coloring) of the nasal cavity at necropsy.

*IN-ND dosing*. The animals were placed in supine position with the head at a 45° angle. A volume of 30 µL (15 µL per nostril) was pipetted at a depth of 1–2 mm. In two studies, the volume was 50 µL, pipetted in one nostril (IN-ND 50 µL).

*Blood sampling*. Blood (15 µL) was taken from the tail vein with the use of EDTA coated microcapillaries (Vitrex Medical^®^, End-to-End Pipettes 15 µL-20 mm, EDTA 3.3 mg/mL), which were put in a micronic tube and stored at −20 °C until analysis.

*Brain sampling*. After decapitation, the skin was removed from the skull, which was then cut along the medial axis until 2–3 mm rostral from the bregma. The parietal and frontal bones were pulled aside or removed with forceps. Care was taken to not cut any further than 2–3 mm rostral from the bregma to ensure no contact with the ethmoid sinus in order to avoid contamination of the samples (see Section 3: we found that the technique for brain sampling differed slightly for one technician, which significantly impacted the results). The olfactory bulbs and the rest of the brain (cerebrum + cerebellum) were collected separately. All the samples were rinsed with PBS and briefly dried before being weighted and stored at −20 °C until analysis.

### 2.2. Test compounds

A chemically diverse set of commercial and internal small molecules spanning a broad physicochemical space in terms of MW, chromatographic hydrophobicity index (CHI) Log*D* (Valkó et al., 1997) and EPSA (Goetz et al., 2014) were tested. Table 1 gives an overview of these compounds, the different formulations and routes tested. Additional physicochemical properties can be found in Supplementary data (1°). The molecular weight goes from 237 (JNJ-04) to 512 (JNJ-02), hence well within drug-like space. Chromatographic hydrophobicity index Log*D*_7.4_ values ranged from −0.09 for ciprofloxacin, a peripherally restricted antibiotic, to 2.6 for JNJ-06. The chromatographic Log*P* values went from −0.03 for ciprofloxacin to 3.47 for JNJ-01. Finally, EPSA values, which have been demonstrated to correlate well with intrinsic permeability (Goetz et al., 2014), ranged from 47 for lidocaine to 112 for domperidone.

**Table 1.** Compounds, formulations, and routes tested.

Compound	Formulation	Routes
Domperidone	40% SBEβCD	IV, IN-NTB
Lidocaine	McIlvaine buffer	SC, IN-NTB, IN-ND 50 µL
Ciprofloxacin	40% SBEβCD	IV, IN-NTB
Minoxidil	40% HPβCD40% SBEβCD	IV, IN-NTB
Morphine	McIlvaine buffer	IV, SC, IN-NTB, IN-ND 50 µL, IN-ND
JNJ-01	20% PEG 40% SBEβCD McIlvaine buffer	IV, IN-NTB
JNJ-02	40% SBEβCD	IV, IN-NTB
JNJ-03	20% SBEβCD 40% PEG400 40% SBEβCD	IV, IN-NTB
JNJ-04	0,9% NaCl 40% SBEβCD	IV, IN-NTB
JNJ-05	40% SBEβCD	IV, IN-NTB
JNJ-06	40% SBEβCD	IV, IN-NTB

SBEβCD: sulfobutylether β-cyclodextrin; HPβCD: hydroxypropyl β-cyclodextrin; PEG: polyethylene glycol 400 monooctylether.

Citric acid monohydrate, di-sodium hydrogen phosphate dehydrate, and ethanol were purchased from Merck Millipore (Brussels, Belgium). Captisol (SBEβCD, sulfobutylether β-cyclodextrin) was obtained by Cydex Pharmaceuticals (Lawrence, KS). PEG400 (polyethylene glycol 400 monooctylether) was purchased by Acros Organics (Geel, Belgium). Lidocaine was obtained from Sigma (Darmstadt, Germany, now Merck, Darmstadt, Germany). JNJ compounds were available from the Janssen compound collection. Morphine [N-methyl-^3^H] was obtained by Biotrend (Köln, Germany).

McIlvaine buffers were prepared using different proportions of 0.1 M citric acid and 0.2 M disodium hydrogen phosphate to get pH 4 or pH 6 solutions. Twenty percent PEG, 20% SBEβCD, or 40% SBEβCD were then dissolved in the selected buffer to obtain the desired formulation.

### 2.3. Bioanalysis of blood and tissue samples

*Sample preparation*. Blood samples were prepared by protein precipitation. More precisely, 15 µL of blood was mixed with 15 µL of H_2_O, 15 µL of DMSO (with or without (internal) standard), and 300 µL of acetonitrile. After vortex and centrifugation, an aliquot, viz. 0.5–2 μL, of the supernatant was injected into the LC–MS/MS system. Tissue samples were homogenized in water (1/10 ratio; v/v) and to 50 µL of homogenate, 50 µL of DMSO and 400 µL of acetonitrile was added. After vortex and centrifugation, an aliquot, viz. 0.5–2 µL, was injected into the LC–MS/MS system.

*UPLC–MS/MS analysis*. Liquid chromatography was mostly performed with a Waters Acquity BEHC18 (2.1 × 50 mm; 1.7 μm particles) column maintained at 55 °C on a Waters Acquity UPLC (Waters, Milford, MA). The autosampler was maintained at 15 °C to prevent water condensation in the autosampler compartment. The mobile phases consisted of H_2_O + 0.1% of formic acid (eluent A) and acetonitrile (eluent B) in most cases. In some case though, a basic mobile phase A was used, viz. 0.01 M bicarbonate in H_2_O (eluent A). Semi-high throughput chromatography was accomplished using a ‘steep’ gradient from 90% A to 10% A in 1.1 minute followed by return to initial conditions and conditioning for another 0.6 min, resulting in an overall run time of 1.7 min. The flow rate was 600 µL/min. Gradient conditions were adapted per compound to have the compound elute between 0.6 and 1 min.

MS/MS analysis was performed using a Sciex 4000 or 5000 QqQ mass spectrometer (Sciex, Framingham, MA) mostly operating in positive ion mode using electrospray ionization. In order to obtain accurate quantitative data, MS instrument settings were optimized by infusing a separate solution of each compound. Next to source setting like, e.g. ESI voltage, gas flow, temperature, and de-clustering potential, settings like collision energy and collision gas pressure were optimized for optimal S/N ratios and adequate LLOQs. Samples were quantified against calibration curves prepared to cover the concentration range of the study samples. The curves were prepared in the same matrix as the study samples. If available stable isotope labeled internal standards were used to compensate for potential ion suppression and sample effects. Peak detection, integration, and quantification were accomplished using Analyst Software (Sciex, Framingham, MA).

### 2.4. Visualization of the distribution of morphine

Autoradiography was used to visualize the distribution of morphine in the olfactory bulb and brain. Ten μCi of [^3^H]-morphine was administered to rats (*n* = 5 per time point, per dose route) via the described IN-NTB and IN-ND technique. At 5 or 20 min after dosing, brains were collected with the olfactory bulbs attached and sectioned.

### 2.5. Data analysis

Concentration data of the animals were analyzed for statistical significance using R statistical software with ‘emmeans’ (Estimated Marginal Means, aka Least-Squares Means, R package version 1.2.3) extension package, by performing a one-way analysis of variance (ANOVA) between IN-NTB and IV/SC routes, followed by a post hoc Dunnett's or *t*-test. Differences among the routes or studies were considered statistically significant when *p*<.05.

## 3. Results

### 3.1. *In vivo* model optimization

The IN-NTB administration technique was optimized in terms of depth (tested at 2, 2.3, and 2.5 cm) and volume (10, 15, 20, 25, and 50 µL was tested) using methylene blue solutions. The evaluation was done by visual inspection of the deposition and spreading of the blue coloring (images available on request). There was a clear targeting of the olfactory epithelium using a depth of 2 cm. The coloring extended to the respiratory epithelium and the pharyngeal region when using volumes higher than 20 µL and occasionally leakage from the nose was seen when using 50 µL. The standardized IN-NTB technique we decided to use, is summarized as follows:

- 2 Fr narrowing to tip of 1.2 Fr (0.4 mm) catheter;
- Depth: 2 cm;
- Volume: 15 µL/nostril;
- Infusion over a period of 60 s/nostril (first right then left) by infusion pump at 0.9 mL/h.

### 3.2. Compound concentrations in blood and tissues

The compound concentrations in blood (*C*_bl_), the olfactory bulbs (*C*_OB_), and the rest of the brain (cerebrum + cerebellum, *C*_br_) were used to calculate blood area under the curve (AUCs) (0 to last time point) and several ratios (*C*_br_/*C*_bl_, *C*_OB_/*C*_bl_, *C*_br_/AUC_bl_ _0–last_, *C*_OB_/AUC_bl_ _0–last_) on an individual animal level, which were then averaged. We used a calculation with blood AUCs to also include potential influences of the systemic exposure preceding sample taken at sacrifice. These values were used to assess reproducibility and finally to assess the possible advantage, i.e. resulting in a higher ratio, of IN delivery compared to IV or SC administration. We chose to limit the number of end points in this screening model; therefore, AUC calculations for tissues were not possible. Hence, we did not calculate other derivatives reported in literature such as drug targeting index (DTI), direct transport percentage (DTP) (Wang et al., 2003; Zhang et al., 2004) or relative contribution of the direct pathway (RC) (Lee et al., 2010).

#### 3.2.1. Reproducibility

At a certain point, we came across some unexpected results: the tissue concentrations of compound JNJ-02 and JNJ-03 differed highly between studies (Supplementary data, 2°). The compounds were dosed by one technician in the different studies, but the sampling was performed by different technicians. After evaluation of the performed procedures, it appeared that sampler 1 used a slightly different technique: the skull was cut slightly further rostral to obtain a better view on the olfactory bulbs. We believe that this action resulted in contamination from the dosing site via the ethmoid sinus. Consequently, all results from sampler 1 were excluded from the data analysis.

**Figure 1.**
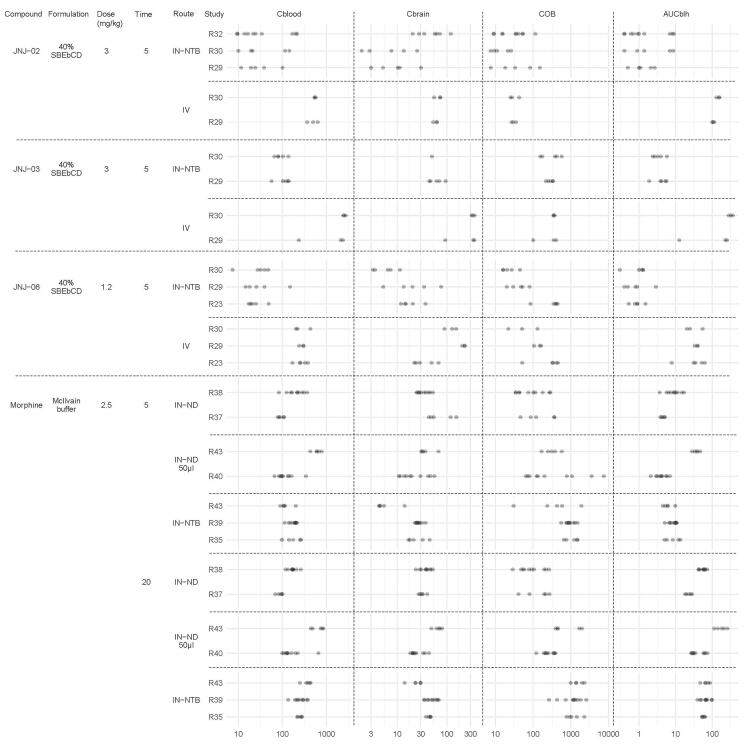
Individual animal blood (ng/mL) and tissue (ng/g) compound concentrations and AUC_bl_ values (h.ng/mL) to visualize the variation between repeated studies.

Only morphine, JNJ-02, JNJ-03, and JNJ-06 were tested in multiple studies. Table 2 summarizes the mean values of the different ratios per study. The *p* levels and graphical representations of individual ratios can be found in Supplementary data (3° and 4°, respectively). The results show that variability is high and reproducibility is not uniform even when the repeated studies are performed by the same sampler (indicated as *n* = 1 sampler in Supplementary data 3°). To give an idea on the absolute blood and tissue concentration range and variation, the individual animal data are visualized in Figure 1.

**Table 2.** Compounds repeated in multiple studies (indicated by study numbers): mean values of the different ratios (+ standard deviation, SD; number of values, *n*) per study.

Compound	Time point (min)	Route	Study	*C*_br_/*C*_bl_			*C*_br_/AU*C*_bl 0-last_			*C*_OB_/*C*_bl_			*C*_OB_/AUC_bl 0-last_		
				Mean	SD	*n*	Mean	SD	*n*	Mean	SD	*n*	Mean	SD	*n*
Morphine	5	IN-ND	R37	0.97	0.70	5	19.46	13.34	5	2.069	1.60	5	43.27	34.66	5
			R38	0.18	0.066	10	4.14	1.55	10	0.59	0.6	10	13.53	13.31	10
		IN-ND 50 µL	R40	0.22	0.16	10	6.56	4.54	10	13.80	25.93	10	391.77	744.23	10
			R43	0.068	0.029	5	1.10	0.41	5	0.56	0.25	5	9.66	6.29	5
		IN-NTB	R35	0.15	0.049	5	3.092	0.80	5	6.55	2.34	5	138.73	57.22	5
			R39	0.16	0.065	10	3.39	1.23	10	5.77	2.46	10	121.52	59.042	10
			R43	0.05	0.012	5	0.98	0.27	5	4.37	3.43	5	88.98	74.28	5
	20	IN-ND	R37	0.37	0.059	5	1.43	0.24	5	1.78	0.98	5	7.021	4.27	5
			R38	0.23	0.051	10	0.69	0.14	10	0.67	0.48	10	2.027	1.41	10
		IN-ND 50 µL	R40	0.17	0.055	10	0.65	0.16	10	1.89	0.97	10	7.22	3.69	10
			R43	0.10	0.017	5	0.40	0.14	5	1.61	1.34	5	5.54	4.17	5
		IN-NTB	R35	0.18	0.033	5	0.78	0.11	5	5.31	2.97	5	22.78	12.11	5
			R39	0.2	0.031	10	0.78	0.13	10	5.11	3.71	10	19.46	13.079	10
			R43	0.065	0.005	5	0.35	0.031	5	4.55	1.65	5	24.27	8.41	5
JNJ-02	5	IN-NTB	R29	0.39	0.31	5	9.67	7.51	5	3.24	5.54	5	76.34	126.76	5
			R30	0.26	0.29	5	5.78	7.57	5	0.81	0.92	5	16.66	21.92	5
			R32	3.46	4.46	7	81.61	104.55	7	2.42	3.83	10	56.89	89.77	10
		IV	R29	0.13	0.044	3	0.56	0.088	3	0.063	0.015	3	0.28	0.02	3
			R30	0.12	0.016	3	0.47	0.096	3	0.057	0.015	3	0.22	0.053	3
JNJ-03	5	IN-NTB	R29	0.65	0.33	5	18.12	10.31	5	2.89	1.34	5	80.34	43.56	5
			R30	0.62	–	1	15.15	–	1	4.00	2.61	5	103.47	66.23	5
		IV	R29	0.23	0.14	3	3.43	3.42	3	0.25	0.15	3	3.71	3.68	3
			R30	0.13	0.017	3	1.06	0.22	3	0.14	0.013	3	1.11	0.19	3
JNJ-06	5	IN-NTB	R23	0.76	0.10	5	21.56	5.51	5	14.83	8.65	5	422.25	262.96	5
			R29	0.74	0.37	5	29.32	13.86	5	1.56	1.23	5	59.18	42.96	5
			R30	0.33	0.38	5	8.97	9.05	5	1	0.66	5	28.54	16.28	5
		IV	R23	0.15	0.09	5	1.86	2.48	5	1.25	0.90	5	16.39	23.41	5
			R29	0.80	0.048	3	5.88	0.25	3	0.50	0.052	3	3.67	0.47	3
			R30	0.48	0.21	3	4.31	1.94	3	0.21	0.097	3	1.83	0.68	3

Highlighted values: *p*<.05, suggesting different outcomes between the two or three repeat studies (per compound, time point and route).

*Morphine*: Several IN dosing routes were repeated (IN-ND, IN-ND 50 µL: two studies; IN-NTB: three studies). The brain ratios were significantly different between studies for all dosing routes (except the *C*_br_/*C*_bl_ ratio at 5 min after IN-ND 50 µL), although in some cases the ratios did not differ even a twofold (e.g. at 20 min after IN-ND (*C*_br_/*C*_bl_ 0.37 vs. 0.23) and IN-ND 50 µL (*C*_br_/*C*_bl_ 0.17 vs. 0.10)). The OB ratios were only significantly different after IN-ND dosing. Despite the ratios 5 min after IN-ND 50 µL differed more than 10-fold, they were not calculated as significant.

*JNJ-02*: Two studies were performed with both the IV and IN-NTB routes, only the IN-NTB route was repeated in a third study. There were no significant differences, even though the brain ratios from the third study were remarkably higher (*C*_br_/AUC_bl_ 81.61 vs. 9.67 and 5.78).

*JNJ-03*: Two studies were performed with both the IV and IN-NTB routes. There were no significant differences. However, there was a threefold difference in the AUC_bl_ ratios after IV dosing. The individual plots (Figure 1) indeed show similar tissue concentrations but differing AUCs (values in study R30 higher than in R29).

*JNJ-06*: Three studies were performed with both the IV and IN-NTB routes. After IN-NTB dosing all ratios, except *C*_br_/*C*_bl_, were significantly different. Unexpectedly, the *C*_br_/*C*_bl_ ratios were also significantly different after IV dosing, which probably originated from differing brain concentrations (Figure 1).

Apart from the sampling technique, we have not statistically identified other potential variables that might account for this difference and it was not possible to leave out a specific sampler (other than sampler 1) neither was it possible to exclude a specific study. Hence, we decided to evaluate the data from these compounds per study as well as grouped over studies, to assess potential advantage of IN dosing by comparing routes.

#### 3.2.2. Comparison of routes

As mentioned previously, concluding that direct NTB transport might be involved, is based on the comparison of an IN dosing route versus a systemic route (IV or SC) by using the calculated ratios. A higher tissue/blood ratio would mean a possible advantage, whether this would be due to a higher tissue concentration or a lower systemic exposure. Based on the significance of at least one of the ratios, we concluded a possible advantage of the nasal route.

First, we evaluated this comparison for the compounds that have been repeated, to see if the same conclusion would be made per study. Table 3 summarizes the same data from Table 2 in a different manner, grouped per study, the corresponding *p* levels can be found in Supplementary data (5°). Although IN dosing routes with morphine were tested multiple times, the IV and SC routes were not repeated; hence, the route comparison for morphine is only given grouped over studies (see further, Table 4).

**Table 3.** Mean values of the different ratios (+ standard deviation, SD; number of values, *n*) per study.

Compound	Time point (min)	Study	Route	*C*_br_/*C*_bl_			*C*_br_/AUC_bl 0-last_			*C*_OB_/*C*_bl_			*C*_OB_/AUC_bl 0-last_		
				Mean	SD	*n*	Mean	SD	*n*	Mean	SD	*n*	Mean	SD	*n*
JNJ-02	5	R29	IN-NTB	0.39	0.31	5	9.67	7.51	5	3.24	5.54	5	76.34	126.76	5
			IV	0.13	0.044	3	0.56	0.088	3	0.063	0.015	3	0.28	0.02	3
		R30	IN-NTB	0.26	0.29	5	5.78	7.57	5	0.81	0.92	5	16.66	21.92	5
			IV	0.12	0.016	3	0.47	0.096	3	0.057	0.015	3	0.22	0.053	3
JNJ-03	5	R29	IN-NTB	0.65	0.33	5	18.12	10.31	5	2.89	1.34	5	80.34	43.56	5
			IV	0.23	0.14	3	3.43	3.42	3	0.25	0.15	3	3.71	3.68	3
		R30	IN-NTB	0.62	–	1	15.15	–	1	4.00	2.61	5	103.47	66.23	5
			IV	0.13	0.017	3	1.06	0.22	3	0.14	0.013	3	1.11	0.19	3
JNJ-06	5	R23	IN-NTB	0.76	0.10	5	21.56	5.51	5	14.83	8.65	5	422.25	262.96	5
			IV	0.15	0.09	5	1.86	2.48	5	1.25	0.90	5	16.39	23.41	5
		R29	IN-NTB	0.74	0.37	5	29.32	13.86	5	1.56	1.23	5	59.18	42.96	5
			IV	0.80	0.048	3	5.88	0.25	3	0.50	0.052	3	3.67	0.47	3
		R30	IN-NTB	0.33	0.38	5	8.97	9.05	5	1	0.66	5	28.54	16.28	5
			IV	0.48	0.21	3	4.31	1.94	3	0.21	0.097	3	1.83	0.68	3

This table is only a different presentation of Table 2, ameliorating visual comprehensibility: the data are grouped per study for each compound so that the dosing routes can be compared more easily. Highlighted values: *p*<.05, compared to the systemic route in the corresponding study.

**Table 4.** Mean values of the different ratios (+ standard deviation, SD; number of values, *n*) grouped over studies.

Compound	Formulation	Dose (mg/kg)	Time point (min)	Route	*C*_br_/*C*_bl_			*C*_br_/AUC_bl 0-last_			*C*_OB_/*C*_bl_			*C*_OB_/AUC_bl 0-last_		
					Mean	SD	*n*	Mean	SD	*n*	Mean	SD	*n*	Mean	SD	*n*
Domperidone	40% SBEbCD	3	5	IN-NTB	0.91		1	13.78		1	39.57		1	596.51		1
				IV	0.04	0.007	5	0.2	0.039	5	0.32	0.075	2	1.69	0.31	2
Lidocaine	McIlvaine buffer	3	5	IN-ND 50 µL	6.42	1.26	10	81.6	31.43	10	5.56	0.88	10	70.40	24.16	10
				IN-NTB	5.32	0.64	5	79.5	19.93	5	6.91	1.16	5	103.62	28.50	5
				SC	12.70	6.04	5	322.96	186.97	5	11.13	5.31	5	282.98	163.39	5
			20	IN-ND 50 µL	3.92	0.51	10	11.22	4.07	10	3.65	0.58	10	10.46	3.96	10
				IN-NTB	3.89	0.52	5	12.18	3.73	5	4.07	0.52	5	12.70	3.62	5
				SC	3.72	0.84	5	11.14	4.33	5	3.1	0.68	5	9.24	3.5	5
Ciprofloxacin	40% SBEbCD	3	5	IN-NTB	0.68	0.12	2	15.83	0.94	2	38.77	58.53	5	871.97	1270.1	5
				IV	0.04	0.003	5	0.21	0.026	5	0.06	0.012	5	0.32	0.07	5
Minoxidil	40% HPβCD	3	5	IN-NTB	0.18	0.046	5	4.22	1.17	5	3.63	1.52	5	85.38	37.72	5
				IV	0.058	0.007	5	0.47	0.14	5	0.06	0.006	5	0.52	0.11	5
	40% SBEbCD	3	5	IN-NTB	0.189	0.087	5	4.62	1.67	5	12.04	13.06	5	307.71	363.55	5
				IV	0.063	0.008	5	0.41	0.035	5	0.07	0.013	5	0.47	0.069	5
Morphine	McIlvaine buffer	2.5	5	IN-ND	0.44	0.54	15	9.25^b^	10.4	15	1.09	1.22	15	23.45	25.84	15
				IN-ND 50 µL	0.17	0.15	15	4.74	4.52	15	9.39	21.77	15	264.40	625.17	15
				IN-NTB	0.13	0.07	20	2.71	1.39	20	5.61	2.68	20	117.69	62.00	20
				IV	0.29	0.13	3	1.33	0.37	3	0.35	0.22	3	1.53	0.65	3
				SC	0.10	0.024	5	1.81	0.58	5	0.1	0.03	5	1.83	0.64	5
			20	IN-ND	0.27^a,b^	0.088	15	0.93^a^^,^^b^	0.4	15	1.04	0.84	15	3.69	3.53	15
				IN-ND 50 µL	0.15^a^	0.056	15	0.56	0.19	15	1.8	1.07	15	6.66	3.79	15
				IN-NTB	0.16^a^	0.063	20	0.67	0.22	20	5.02^a,b^	3	20	21.49^a,b^	11.46	20
				IV	0.40	0.082	3	0.52	0.047	3	0.43	0.057	3	0.57	0.051	3
				SC	0.19	0.025	5	0.39	0.051	5	0.2	0.023	5	0.41	0.064	5
JNJ-01	20% PEG	5	5	IN-NTB	0.81	0.40	5	30.17	14.22	5	1.62	0.58	5	60.44	22.14	5
				IV	9.84	0.78	3	84.67	19.58	3	8.65	1.03	3	74.84	20.56	3
			20	IN-NTB	0.26	0.044	5	1.28	0.26	5	0.58	0.24	5	2.70	0.92	5
				IV	7.24	2.63	3	12.68	2.05	3	6.05	1.64	3	10.74	0.95	3
	40% SBEbCD	5	5	IN-NTB	0.84	0.97	5	29.93	36.08	5	0.9	0.62	5	31.70	23.77	5
				IV	7.09	2.84	3	60.75	36.42	3	6.51	2.13	3	55.27	29.08	3
			20	IN-NTB	0.32	0.14	5	1.58	0.6	5	0.53	0.14	5	2.52	0.41	5
				IV	6.38	1.79	3	12.92	4.63	3	4.89	1.1	3	9.86	3	3
	McIlvaine buffer	5	5	IN-NTB	0.26	0.076	5	8.39	1.75	5	0.52	0.18	5	18.14	8.66	5
				IV	11.30	2.17	3	82.23	22.05	3	10.93	1.89	3	79.21	17.79	3
			20	IN-NTB	0.28	0.13	5	1.29	0.49	5	0.97	0.49	5	4.68	2.30	5
				IV	9.33	0.48	3	17.06	3.67	3	7.92	0.65	3	14.46	3.12	3
JNJ-02	40% SBEbCD	3	5	IN-NTB	0.33	0.29	10	7.72	7.4	10	2.02	3.96	10	46.50	91.34	10
				IV	0.13	0.029	6	0.51	0.098	6	0.06	0.014	6	0.25	0.052	6
JNJ-03	20% SBEbCD	1.2	5	IN-NTB	0.39	0.28	5	8.29	5.86	5	7.65	10.01	5	176.95	242.14	5
				IV	0.17	0.024	5	1.8	0.49	5	0.18	0.025	5	1.89	0.53	5
	40% PEG400	0.25	5	IN-NTB	0.30	0.08	5	5.71	2.2	5	1	0.17	5	18.93	5.32	5
				IV	0.24	0.051	5	2.56	0.69	5	0.24	0.017	5	2.50	0.35	5
	40% SBEbCD	0.25	5	IN-NTB	2.66	2.79	2	48.83	45.15	2	7.88	7.09	5	147.34	124.02	5
				IV	0.08	0.026	4	0.76	0.17	4	0.09	0.041	4	0.84	0.27	4
		1.2	5	IN-NTB	0.57	0.68	5	13.6	15.81	5	2.62	2.45	5	57.75	41.71	5
				IV	0.10	0.02	5	1.01	0.24	5	0.13	0.021	5	1.29	0.30	5
		3	5	IN-NTB	0.65	0.29	6	17.62	9.3	6	3.45	2.04	10	91.90	54.24	10
				IV	0.18	0.10	6	2.24	2.53	6	0.2	0.11	6	2.41	2.73	6
JNJ-04	0.9% NaCl	3	5	IN-NTB	5.92	1.65	5	84.38	26.2	5	8.21	3.34	5	119.50	59.19	5
				IV	7.66	5.03	5	106.6	86.09	5	5.88	2.07	5	82.03	40.14	5
	40% SBEbCD	3	5	IN-NTB	6.45	1.29	5	166.33	50.73	5	17.06	5.66	5	417.27	97.87	5
				IV	2.00	1	5	12.32	7.08	5	3	1.84	5	18.99	13.22	5
JNJ-05	40% SBEbCD	2.4	30	IN-NTB	0.51	–	1	0.61	–	1	3.61	6.37	4	4.49	7.45	4
				IV	0.65	0.27	5	0.69	0.26	5	0.08	0.014	5	0.09	0.017	5
			60	IN-NTB			0			0	0.74	0.35	2	0.63	0.22	2
				IV	0.73	0.12	5	0.46	0.091	5	0.091	0.038	4	0.053	0.017	4
			180	IN-NTB	0.60	–	1	0.12	–	1	0.55	0.13	4	0.15	0.028	4
				IV	1.18	0.28	5	0.26	0.068	5	0.15	0.02	5	0.03	0.006	5
			360	IN-NTB	5.83	8.42	3	1.01	1.51	3	0.89	0.45	5	0.15	0.085	5
				IV	1.11	0.27	5	0.13	0.038	5	0.18	0.023	5	0.02	0.002	5
JNJ-06	40% SBEbCD	1.2	5	IN-NTB	0.61	0.36	15	19.95	12.74	15	5.8	8.11	15	169.99	233.70	15
				IV	0.42	0.30	11	3.63	2.54	11	0.76	0.75	11	8.95	16.45	11

Highlighted values: significantly (*p*<.05) higher or lower value compared to the non-IN route (morphine: ^a^compared to IV, ^b^compared to SC).

*JNJ-02*: in none of the studies there were significantly higher ratios for the IN-NTB route; hence, we concluded no direct transport. However, when looking at the actual values, we do see large route differences (e.g. study R29: a *C*_OB_/*C*_bl_ ratio of 3.24 after IN-NTB dosing vs. 0.063 after IV dosing); although, this is not calculated as significant. This trend is seen in both studies and made us doubt the conclusion.

*JNJ-03*: in both studies, all the ratios are higher after IN-NTB dosing than IV; however, only the OB ratios are significantly higher in the first study. In the second study, all ratios are significantly higher after IN-NTB dosing. In both studies, we concluded possible direct transport.

*JNJ-06*: in all three studies there was at least one of the ratios significantly higher after IN-NTB dosing. The conclusion, suggesting direct transport, counts for all studies even though the number and type of significant ratios differ. Also, some ratios are much higher after IN-NTB but not calculated as significant (e.g. R29: *C*_OB_/AUC_bl_ 59.18 vs. 3.67).

Next, we evaluated the data of the compounds that were not repeated and the grouped data from the repeated compounds. Table 4 summarizes the mean values of the ratios and the corresponding *p* values can be found in Supplementary data (6°). The results are as follows:

*Domperidone*. All ratios are higher after NTB dosing suggesting a direct brain transport; however, there are only data from one animal (tissue concentrations of 4/5 animals were below quantification limit).

*Lidocaine*. At 20 min, the *C*_OB_/AUC_bl_ ratio is significantly higher after IN-NTB dosing; hence, potential direct transport can be concluded. However, the actual values are not relevantly different. Hence, this conclusion might be doubted (see Section 4).

*Ciprofloxacin*. Both of the *C*_br_ ratios are significantly higher after IN-NTB dosing. The *C*_OB_ ratios are also higher, however, not significant. We conclude a possible direct brain transport.

*Minoxidil*. Two different formulations were tested. All ratios are higher after IN-NTB dosing but only significant for the *C*_br_ ratios and for the *C*_OB_ ratios of the 40% HPβCD formulation. We conclude a possible direct brain transport.

*Morphine*. Each IN route was compared to the SC as well as the IV route. At 5 and 20 min, all ratios except *C*_br_/*C*_bl_ are higher for IN routes compared to SC and IV routes, where only a few are significant. The *C*_br_/*C*_bl_ ratio shows mixed results; only after IN-ND dosing, the ratio is higher than the IV and SC route at 5 min but not significant. We conclude a possible direct brain transport.

*JNJ-01*. Three formulations were tested. None of the ratios after the IN route are higher than IV. We conclude no evidence for direct transport.

*JNJ-02*. All ratios are higher after IN-NTB than after IV but none are significant; hence, we conclude no direct transport. However, looking at the actual values (3- to 15-fold differences for brain ratios); in this case, the conclusion can be doubted (see section 4).

*JNJ-03*. Three formulations, of which one at three doses, were tested. All ratios are higher after IN-NTB than after IV dosing but only a few are significant. Only the lowest dose of the 40% SBEβCD formulation does not have at least one significantly different ratio. We conclude a possible direct transport.

*JNJ-04*. Two formulations were tested. All the ratios from the 40% SBEβCD group are significantly higher after IN-NTB compared to IV. In the 0.9% NaCl group, only the OB ratios are higher after IN, but not significant. We conclude a possible direct transport.

*JNJ-05*. This compound was evaluated over a longer period (30, 60, 180, and 360 min). The brain ratios are non-significantly higher after IN-NTB only at 360 min. The OB ratios are higher at all time points. Significance is only seen from 60 min onwards. This is an example of where a slower direct transportation mechanism might be involved in the delivery to the brain.

*JNJ-06*. All ratios are higher after IN-NTB but only significant when the AUC was used. We conclude a possible direct transport.

The resulting conclusion of the grouped data corresponds with those of the individual studies.

### 3.3. Visualization of the distribution of morphine

**Figure 2.**
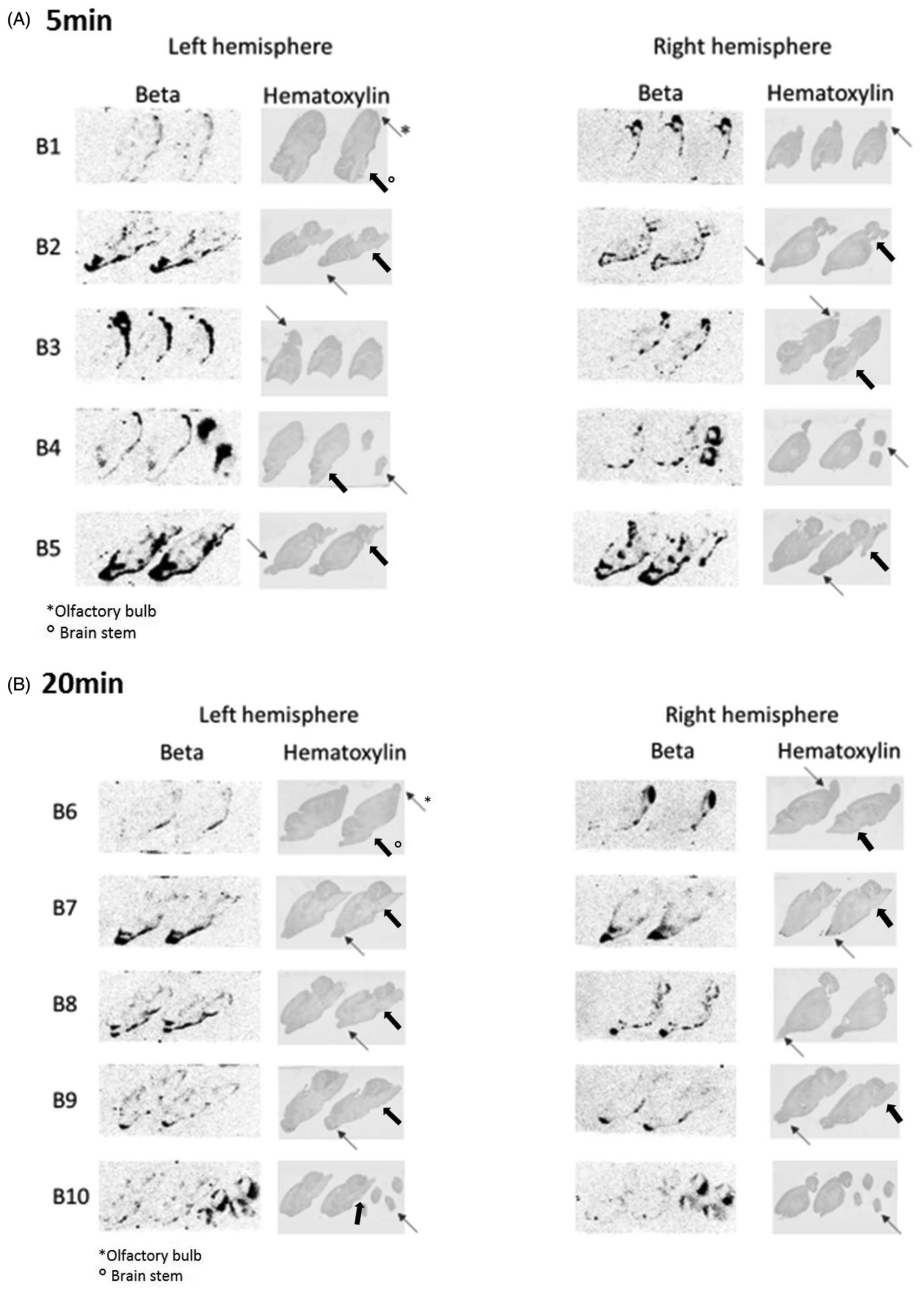
Distribution of [3H]-morphine in rat brain 5 (A) and 20 (B) min after IN-NTB dosing (10 µCi/rat). The figure illustrates autoradiography images of left and right hemisphere of 10 individual animals (B1–10, 5 per time point), including hematoxylin counterstain on the same sections. The thin arrow indicates the olfactory bulb, the wide arrow indicates the brain stem.

Autoradiography revealed clear morphine uptake in the olfactory bulb of all animals at 5 and 20 min after IN-NTB dosing. Other brain regions did not show much presence of morphine, except the area of the olfactory tract and the brain stem in a subset of animals (Figure 2). Similar results were obtained with IN-ND dosing (see Supplementary data, 7°).

## 4. Discussion

Intranasal drug delivery has been intensively investigated the past decades, as it is seen as an easy and feasible noninvasive dosing route for targeting the CNS bypassing the BBB. We are aware that the translational value for this model is uncertain regarding bioavailability and dosage, due to several factors (nasal anatomy, physiology, etc.), which can be found in literature (Gizurarson, 1990; Illum, 2000; Ugwoke et al., 2005; Vyas et al. 2006; Pires et al., 2009; Lee et al., 2010). Still, we too were interested in its possibilities as a proof of concept and seeked to set up a screening platform for a quick impression of a compound’s ability to travel directly to the brain after IN delivery.

Several reviews have pointed out the difficulties of analyzing and interpreting NTB data. There are no guidelines or standards to evaluate IN dosing data in a globally standardized way. The level of detailed information concerning methodology is very variable among publications which make it very difficult to reproduce such results and even more to summarize, use or compare this data.

It was clear that even in a standardized set-up, making a uniform conclusion is not very straightforward. We used different tissue/plasma ratios to compare an IN and non-IN route to evaluate drug delivery to the brain. Besides the tissue to blood ratio at the time of sacrifice, we included tissue to blood AUC ratios to take into account the total systemic exposure. In some cases, this impacted the conclusion, hence, depending on the chosen type of read-out, the conclusion might be different. The question remains which one is the most accurate. Our conclusion is based on the significance of at least one of four ratios. These can be highly variable between as well as within studies and this might be the cause of non-significant outcomes. A combination of factors including potential slight differences in drug deposition and formulation, individual physiology and sampling technique might be the origin of such variability. Increasing the group size could address this issue. However, when the group size is too large, the model becomes less suitable as a screening model. Our data sometimes show much larger ratios after IN-NTB dosing than IV, which were not calculated as significant. It might be more relevant not only to look at significant differences but also to set an *x*-fold difference in median or mean ratios as threshold which could then be investigated in a larger set of animals to conclude possible direct brain transport. Also, looking into the calculations, being able to use individual ratios gives a better view on the animal variability opposed to using averaged data for tissue AUCs. A way to circumvent this would be using microdialysis, measuring brain extracellular fluid concentrations in an individual animal, but this technique and expertise is not readily available for everyone and less suitable as screening model.

Regarding treatments for CNS disorders, we would want the drug to be in the cerebrum and not only in the olfactory bulbs; hence, we separated the olfactory bulbs from the rest of the brain. In some cases, high concentrations of drug or high tissue to blood ratios were found in the OB, but not in the rest of the brain. The high OB concentrations might cause an overestimation of the direct transport to the cerebrum in case these tissues are not analyzed separately. As a possible amelioration of this model, it might even be better to also divide the cerebrum in specific areas to evaluate specific regional drug delivery. Autoradiography of the brain showed that morphine is mainly present in the olfactory bulb, confirming the findings of Westin et al. (2005) and in some animals also the brain stem, which suggest also the trigeminal nerve as entrance to the brain after IN dosing. The ratios are similar or decreased at a later (20 min) time point; hence, this suggests that morphine is not proceeding further into the cerebrum within this period. Looking at one of the internal compounds (JNJ-05) that was tested at later time points (60, 180, and 360 min), the brain ratios after IN-NTB dosing were higher than IV only at 360 min (although not significant) which suggest a slow transport. Since the same formulation (40% SBEβCD) was used for other compounds, showing higher brain ratios after IN-NTB dosing already at 5 min (minoxidil, JNJ-02 (not significant), JNJ-04 and JNJ-06), this is probably a compound specific property. It is however clear that formulation can have a large impact on kinetics, which is frequently described in literature, as are cyclodextrins known to be absorption enhancers (Ugwoke et al., 2005; Vyas et al., 2006; Pires et al., 2009; Gänger & Schindowski, 2018). This is nicely shown for JNJ-04 for which all ratios are much higher after IN-NTB than IV when using the 40% SBEβCD solution but not when using 0.9% NaCl. The correlation analysis between compound physicochemical properties combined with formulation and the direct brain transport was out of scope of this project but it could be interesting to perform more complex analysis or even apply machine learning on sufficiently large data sets to gather more insights on this.

Despite the variability, we believe to show some evidence for possible direct NTB transport for domperidone (albeit in only one animal), ciprofloxacin, minoxidil, morphine, and several internal compounds. However, only for the tested formulations and time frame and without specifying the target region (OB or cerebrum). The results for morphine and lidocaine correspond with literature, stating direct transport (Westin et al., 2006) and concluding no direct transport (Bagger & Bechgaard, 2004), respectively. From the experimental physicochemical properties of the investigated drugs, we see no correlation between Log*D*_7.4_ or EPSA and possible direct NTB uptake. Arguably, however, there might be a correlation with CHI Log*P* as the compounds with highest values (JNJ-01 and JNJ-02) showed no convincing NTB uptake.

Additional studies are needed to assess further distribution in the brain (preferably by imaging) as well as if potential therapeutic concentrations can be reached by evaluating multiple/variable dosing schemes.

## 5. Conclusions

We have standardized an *in vivo* NTB delivery model in rats with the intention to assess the fast direct brain transport of compounds in a screening platform. Performing the model has taught us that sampling technique is an important variable. The olfactory bulbs should be separated from the rest of the brain and care must be taken not to contaminate the different tissues. Potential direct transport to the brain was concluded for some compounds, of which some were already described in literature. The results are highly variable and in some cases the significance was not reproducible. However, the conclusion remained the same whether the repeated studies were evaluated separately or aggregated. Still, it is important to first evaluate the experimental techniques in reproducibility studies and ideally perform validation studies. To achieve this, we believe there is a need for a comprehensive reference data set, with easily available compounds and formulations. Overall, this model is technically simple and readily available and we see it useful for assessment of alternative dosing of a promising compound where different formulations can be tested in a sufficient set of animals.

Comparing brain concentrations to blood concentration at the time of sacrifice or to total exposure in blood (AUC) could make a difference in the evaluation of brain targeting for some compounds. Further evaluation is needed to determine which ratio is most appropriate. As some reviews have already stated, a more uniform methodology and data analysis could improve the interpretation and value concerning this type of *in vivo* data. Guidelines advising on the minimally required dosing technique/formulation details, the correct nomenclature of the dosage (IN vs. IN-ND or NTB), the type of read-out (qualitative vs. quantitative), the data analysis, and comparison with a reference data set would be helpful in this matter.

## Supplementary Material

Supplemental Material

## Data Availability

The data that support the findings of this study are available from the corresponding author upon reasonable request.
