# Supplementary material for: Direct nose to brain delivery of small molecules: critical analysis of data from a standardized in vivo screening model in rats
Source: Drug Deliv. 2020 Nov 10;27(1):1597–607. doi: 10.1080/10717544.2020.1837291 (PMC7655051; doi:10.1080/10717544.2020.1837291)
Supplement: Supplemental Material [file IDRD_A_1837291_SM6603.zip › Manuscript_NTB_Dhuyvetter_Suppl1.docx]

**Supplementary data**

1° Summary of experimental hydrophobicity measures, formulations, dose, and number of animals per study and per dosing route.

| **Compound** | **MW** | **LogD_7.4_** | **LogP** | **EPSA** | **Formulation** | **Dose (mg/kg)** | **Study** | **Route** | | | | |  |
| --- | --- | --- | --- | --- | --- | --- | --- | --- | --- | --- | --- | --- | --- |
|  |  |  |  |  |  |  |  | **IN-ND** | **IN-ND 50 µL** | **IN-NTB** | **IV** | **SC** | **Total** |
| Domperidone | 425 | 1.54^a^, 3.9^3^ | 2.47^a^, 4.2^3^ | 112 | 40% SBEβCD | 3 | R25 |  |  | 5 | 5 |  | 10 |
| Lidocaïne | 234 | 2.48^a^, 1.6^3^ | 2.85^a^, 2.2^3^ | 47 | McIlvaine buffer | 3 | R41 |  | 20 | 10 |  | 10 | 40 |
| Ciprofloxacin | 331 | -0.09^a^, -1.6^c^ | -0.03^a^, 0.28^[[1]](#endnote-1)^ | 106 | 40% SBEβCD | 3 | R25 |  |  | 5 | 5 |  | 10 |
| Minoxidil | 209 | 0.25^a^ | 1.09^a^, 1.24^[[2]](#endnote-2)^ | 93 | 40% HPβCD | 3 | R26 |  |  | 5 | 5 |  | 10 |
|  |  |  |  |  | 40% SBEβCD | 3 | R25 |  |  | 5 | 5 |  | 10 |
| Morphine | 285 | 0.9^3^ | 0.3^[[3]](#endnote-3)^ | 93 | McIlvaine buffer | 2.5 | R43 |  | 10 | 10 |  |  | 20 |
|  |  |  |  |  |  |  | R40 |  | 20 |  |  |  | 20 |
|  |  |  |  |  |  |  | R39 |  |  | 20 |  |  | 20 |
|  |  |  |  |  |  |  | R38 | 20 |  |  |  |  | 20 |
|  |  |  |  |  |  |  | R37 | 10 |  |  |  |  | 10 |
|  |  |  |  |  |  |  | R35 |  |  | 10 | 6 | 10 | 26 |
| JNJ-01 | 460 | 1.76^a^ | 3.47^a^ | 93 | 20% PEG | 5 | R33 |  |  | 10 | 6 |  | 16 |
|  |  |  |  |  | 40% SBEβCD | 5 | R33 |  |  | 10 | 6 |  | 16 |
|  |  |  |  |  | McIlvaine buffer | 5 | R33 |  |  | 10 | 6 |  | 16 |
| JNJ-02 | 512 | 0.86^a^ | 3.46^a^ | 98 | 40% SBEβCD | 3 | R30 |  |  | 5 | 3 |  | 8 |
|  |  |  |  |  |  |  | R29 |  |  | 5 | 3 |  | 8 |
| JNJ-03 | 393 | 1.07^a^ | 1.52^a^ | 81 | 40% SBEβCD | 3 | R30 |  |  | 5 | 3 |  | 8 |
|  |  |  |  |  |  |  | R29 |  |  | 5 | 3 |  | 8 |
|  |  |  |  |  | 20% SBEβCD | 1.2 | R22 |  |  | 5 | 5 |  | 10 |
|  |  |  |  |  | 40% PEG400 | 0.25 | R22 |  |  | 5 | 5 |  | 10 |
|  |  |  |  |  | 40% SBEβCD | 0.25 | R22 |  |  | 5 | 5 |  | 10 |
|  |  |  |  |  | 40% SBEβCD | 1.2 | R22 |  |  | 5 | 5 |  | 10 |
| JNJ-04 | 237 | 1.63^b,^^[[4]](#endnote-4)^ | 2.18^[[5]](#endnote-5)^ | 48 | 0.9% NaCl | 3 | R23 |  |  | 5 | 5 |  | 10 |
|  |  |  |  |  | 40% SBEβCD | 3 | R23 |  |  | 5 | 5 |  | 10 |
| JNJ-05 | 463 | NT | NT | 92 | 40% SBEβCD | 2.4 | R24 |  |  | 20 | 20 |  | 40 |
| JNJ-06 | 469 | 2.59^a^ | 2.90^a^ | 82 | 40% SBEβCD | 1.2 | R30 |  |  | 5 | 3 |  | 8 |
|  |  |  |  |  | 40% SBEβCD | 1.2 | R29 |  |  | 5 | 3 |  | 8 |
|  |  |  |  |  | 40% SBEβCD | 1.2 | R23 |  |  | 5 | 5 |  | 10 |
|  |  |  |  |  |  |  | Total | 30 | 50 | 185 | 117 | 20 | 402 |

^a^ Chromatographical Hydrophobicity Index ^b^Calculated using pK_a_ value of 7.8 provided in ref 4. ^c^Calculated using pK_a_ values of 10.6 and 8.7 provided in ref 1.

1. 1 Hylton, K.; Sangwan, M.; Mitra, S. Microscale membrane extraction of diverse antibiotics from water, *Anal. Chim. Acta* **2009**, 653, 116–120. [↑](#endnote-ref-1)
2. 2 Hansch, C.; Leo, A.;  Hoekman, D. Exploring QSAR - Hydrophobic, Electronic, and Steric

   Constants. Washington, DC: American Chemical Society, 1995, p. 64. [↑](#endnote-ref-2)
3. 3 Dagenais, C.; Avdeef, A.; Tsinman, O.; Dudley, A.; Beliveau, R. P-glycoprotein deficient mouse in situ blood–brain barrier permeability and its prediction using an in combo PAMPA model. *Eur. J. Pharm. Sci.* **2009**, *38,* 121–137. [↑](#endnote-ref-3)
4. 4 Lan, L.; Hu, B.; Yu, C. pH-resistant titania hybrid organic–inorganic coating for stir bar sorptive extraction of drugs of abuse in urine samples followed by high performance liquid chromatography–ultraviolet visible detection. *J. Chromatogr. A* **2010**, *1217*, 7003-7009. [↑](#endnote-ref-4)
5. 5 Zerara, M.; Brickmann, J.; Kretschmer, R.; Exner, T. E. Parameterization of an empirical model for the prediction of *n*-octanol, alkane and cyclohexane/water as well as brain/blood partition coefficients. *J. Comput. Aided Mol. Des.* **2009**, *23*, 105–111. [↑](#endnote-ref-5)
