# Supplementary material for: Direct nose to brain delivery of small molecules: critical analysis of data from a standardized in vivo screening model in rats
Source: Drug Deliv. 2020 Nov 10;27(1):1597–607. doi: 10.1080/10717544.2020.1837291 (PMC7655051; doi:10.1080/10717544.2020.1837291)
Supplement: Supplemental Material [file IDRD_A_1837291_SM6603.zip › Manuscript_NTB_Dhuyvetter_Suppl2.docx]

**Supplementary data**

2° Compound concentrations (mean ± sd, n=5 unless stated otherwise) in olfactory bulbs (C_OB_) and rest of brain (C_br_) 5 min after dosing. The compounds were dosed by one technician at the same dose (3 mg/kg) in different studies, the sampling was performed by different technicians. The results for sampler 1 differed highly and were excluded from further analysis.

| **Compound** | **Study** | **Sampler** | **C_OB_ (ng/g)** | **C_br_ (ng/g)** |
| --- | --- | --- | --- | --- |
| JNJ-02 | R27 | Sampler 1 | 11222 ± 4408 | 205 ± 87 |
|  | R29 | Sampler 2 | 59 ± 59 | 12 ± 11 |
|  | R30 | Sampler 2 | 15 ± 8 | 10 ± 10 |
|  | R32 | Sampler 1 | 6727 ± 3264 | 335 ± 206 |
|  |  | Sampler 2 | 41 ± 44 | 61 ± 51 (n=3) |
|  |  | Sampler 3 | 35 ± 16 | 53 ± 23 (n=4) |
| JNJ-03 | R27 | Sampler 1 | 10134 ± 9272 | 178 ± 129 |
|  | R29 | Sampler 2 | 286 ± 51 | 64 ± 20 |
|  | R30 | Sampler 2 | 342 ± 177 | 50 (n=1) |
