# Supplementary material for: Direct nose to brain delivery of small molecules: critical analysis of data from a standardized in vivo screening model in rats
Source: Drug Deliv. 2020 Nov 10;27(1):1597–607. doi: 10.1080/10717544.2020.1837291 (PMC7655051; doi:10.1080/10717544.2020.1837291)
Supplement: Supplemental Material [file IDRD_A_1837291_SM6603.zip › Manuscript_NTB_Dhuyvetter_Suppl3.docx]

**Supplementary data**

3° Compounds repeated in multiple studies (indicated by n studies): p-values of the calculated ratios between different studies. Values <0.05 are highlighted and suggest low reproducibility.

| **Compound** | **Formulation** | **Dose**  **(mg/kg)** | **Time point (min)** | **Route** | **n Studies** | **p**  **C_br_/C_bl_** | **p C_br_/AUC_bl_ _0-last_** | **p**  **C_OB_/C_bl_** | **p**  **C_OB_/ AUC_bl_ _0-last_** | **n Samplers** |
| --- | --- | --- | --- | --- | --- | --- | --- | --- | --- | --- |
| Morphine | McIlvain buffer | 2.5 | 5 | IN-ND | 2 | 0.0028 | 0.0025 | 0.020 | 0.029 | 2 |
|  |  |  |  | IN-ND 50µl | 2 | 0.050 | 0.021 | 0.28 | 0.28 | 2 |
|  |  |  |  | IN-NTB | 3 | 0.0039 | 0.0011 | 0.44 | 0.45 | 1 |
|  |  |  | 20 | IN-ND | 2 | <0.001 | <0.001 | 0.010 | 0.0043 | 2 |
|  |  |  |  | IN-ND 50µl | 2 | 0.020 | 0.011 | 0.65 | 0.44 | 2 |
|  |  |  |  | IN-NTB | 3 | <0.001 | <0.001 | 0.92 | 0.74 | 1 |
| JNJ-02 | 40% SBEbCD | 3 | 5 | IN-NTB | 3 | 0.13 | 0.13 | 0.61 | 0.57 | 2 |
|  |  |  |  | IV | 2 | 0.88 | 0.28 | 0.65 | 0.10 | 1 |
| JNJ-03 | 40% SBEbCD | 3 | 5 | IN-NTB | 2 | 0.94 | 0.81 | 0.42 | 0.53 | 1 |
|  |  |  |  | IV | 2 | 0.27 | 0.30 | 0.25 | 0.29 | 1 |
| JNJ-06 | 40% SBEbCD | 1.2 | 5 | IN-NTB | 3 | 0.090 | 0.024 | 0.0014 | 0.0027 | 2 |
|  |  |  |  | IV | 3 | <0.001 | 0.063 | 0.12 | 0.43 | 2 |
