# Supplementary material for: Direct nose to brain delivery of small molecules: critical analysis of data from a standardized in vivo screening model in rats
Source: Drug Deliv. 2020 Nov 10;27(1):1597–607. doi: 10.1080/10717544.2020.1837291 (PMC7655051; doi:10.1080/10717544.2020.1837291)

**Supplementary data**

4° Graphical presentations of the calculated ratios (Y-axis) in different studies (X-axis) to evaluate reproducibility. Graphs per compound, formulation, dose, route and time point (graph headers), colored by sampler.


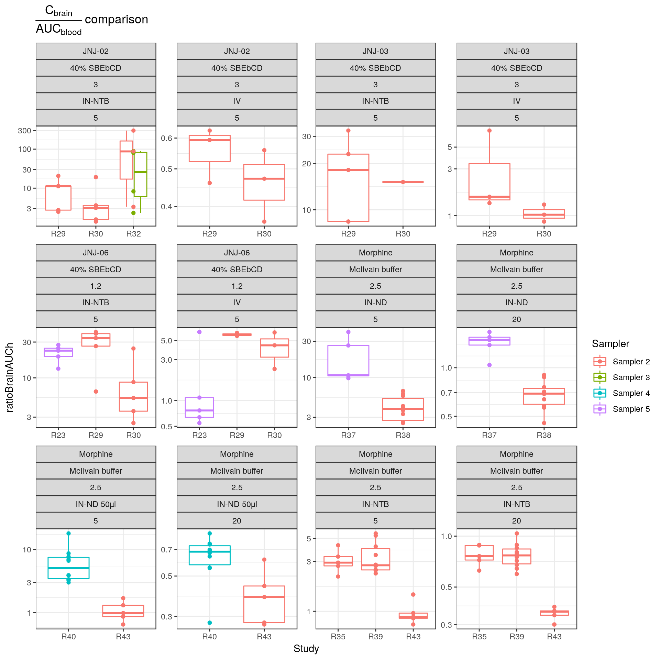

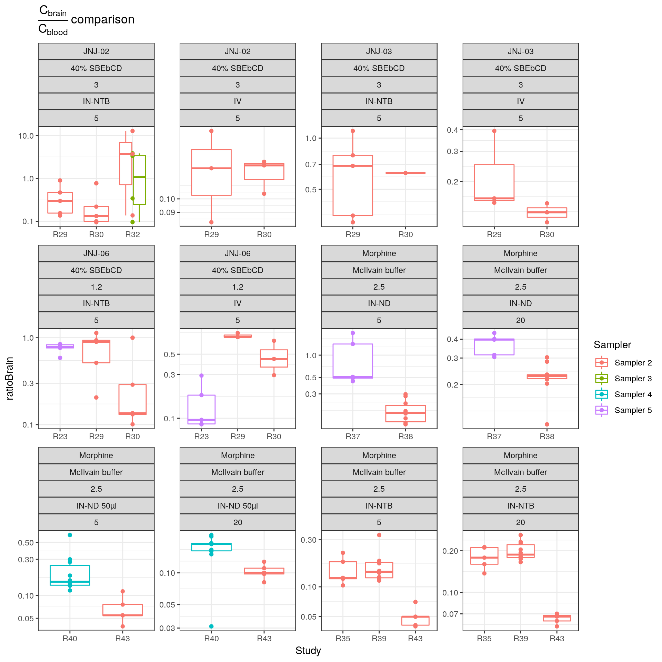


*4° Continued* - Graphical presentations of the calculated ratios (Y-axis) in different studies (X-axis) to evaluate reproducibility. Graphs per compound, formulation, dose, route and time point (graph headers), colored by sampler.


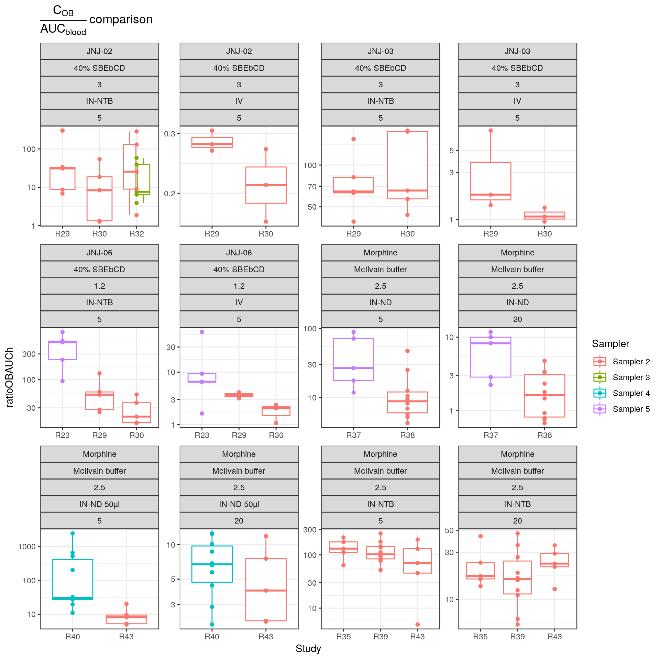

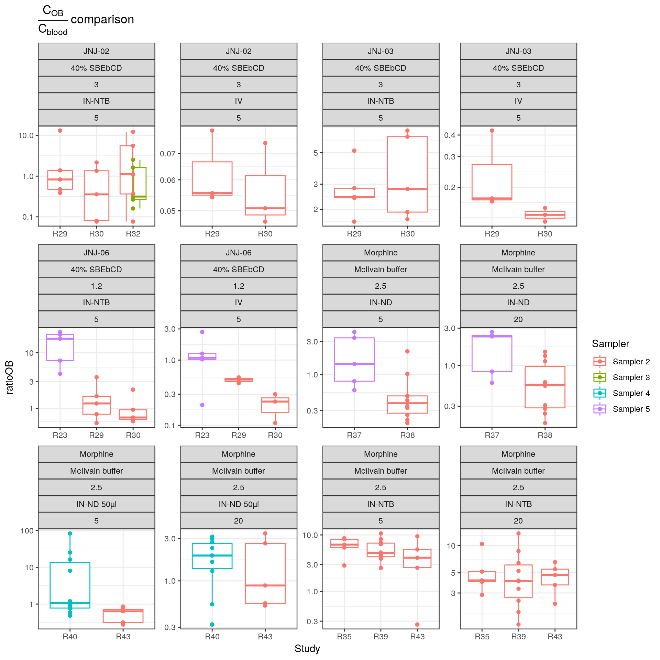

Supplement: Supplemental Material [file IDRD_A_1837291_SM6603.zip › Manuscript_NTB_Dhuyvetter_Suppl4.docx]
