# Supplementary material for: Direct nose to brain delivery of small molecules: critical analysis of data from a standardized in vivo screening model in rats
Source: Drug Deliv. 2020 Nov 10;27(1):1597–607. doi: 10.1080/10717544.2020.1837291 (PMC7655051; doi:10.1080/10717544.2020.1837291)
Supplement: Supplemental Material [file IDRD_A_1837291_SM6603.zip › Manuscript_NTB_Dhuyvetter_Suppl5.docx]

**Supplementary data**

5° Compounds repeated in multiple studies: p-values of the calculated ratios between the different routes, per study. Values <0.05 are highlighted and suggest different outcomes between routes. The route that resulted in the highest ratio is set in bold. If an IN route is set in bold, this suggests an additional direct brain transport.

| **Compound** | **Study** | **Route contrast** | **p**  **C_br_/C_bl_** | **p**  **C_br_/AUC_bl_ 0-last** | **p**  **C_OB_/C_bl_** | **p**  **C_OB_/ AUC_bl_ 0-last** |
| --- | --- | --- | --- | --- | --- | --- |
| JNJ-02 | R29 | IN-NTB - IV | 0.21 | 0.088 | 0.37 | 0.35 |
|  | R30 | IN-NTB - IV | 0.44 | 0.28 | 0.22 | 0.26 |
| JNJ-03 | R29 | **IN-NTB** - IV | 0.086 | 0.059 | 0.017 | 0.026 |
|  | R30 | **IN-NTB** - IV | 0.002 | <0.001 | 0.048 | 0.041 |
| JNJ-06 | R23 | **IN-NTB** - IV | <0.001 | <0.001 | 0.008 | 0.009 |
|  | R29 | **IN-NTB** - IV | 0.80 | 0.030 | 0.20 | 0.073 |
|  | R30 | **IN-NTB** - IV | 0.57 | 0.43 | 0.1 | 0.033 |
