# Supplementary material for: Direct nose to brain delivery of small molecules: critical analysis of data from a standardized in vivo screening model in rats
Source: Drug Deliv. 2020 Nov 10;27(1):1597–607. doi: 10.1080/10717544.2020.1837291 (PMC7655051; doi:10.1080/10717544.2020.1837291)
Supplement: Supplemental Material [file IDRD_A_1837291_SM6603.zip › Manuscript_NTB_Dhuyvetter_Suppl6.docx]

**Supplementary data**

6° P-values of the calculated ratios between different routes. Values <0.05 are highlighted. The route that resulted in the highest ratio is in bold. If an IN route is in bold, this suggests an additional direct brain transport. In case both routes are in bold, the route with the highest ratio is specified between brackets.

| **Compound** | **Formulation** | **Dose (mg/kg)** | **Time point (min)** | **Compared routes** | **p**  **C_br_/C_bl_** | **p**  **C_br_/AUC_bl_ 0-last** | **p**  **C_OB_/C_bl_** | **p**  **C_OB_/ AUC_bl_ 0-last** |
| --- | --- | --- | --- | --- | --- | --- | --- | --- |
| Domperidone | 40% SBEbCD | 3 | 5 | **IN-NTB** - IV | <0.001 | <0.001 | 0.0015 | <0.001 |
| Lidocaine | McIlvain buffer | 3 | 5 | IN-ND 50µl - **SC** | 0.0033 | <0.001 | 0.0031 | <0.001 |
|  |  |  |  | IN-NTB - **SC** | 0.0029 | 0.0015 | 0.047 | 0.0060 |
|  |  |  | 20 | IN-ND 50µl - SC | 0.76 | 1.00 | 0.19 | 0.78 |
|  |  |  |  | **IN-NTB** - SC | 0.85 | 0.88 | 0.035 | 0.29 |
| Ciprofloxacin | 40% SBEbCD | 3 | 5 | **IN-NTB** - IV | <0.001 | <0.001 | 0.18 | 0.16 |
| Minoxidil | 40% HPβCD | 3 | 5 | **IN-NTB** - IV | <0.001 | <0.001 | <0.001 | 0.0010 |
|  | 40% SBEbCD | 3 | 5 | **IN-NTB** - IV | 0.012 | <0.001 | 0.075 | 0.095 |
| Morphine | McIlvain buffer | 2.5 | 5 | IN-NTB - IV | 0.72 | 0.96 | 0.81 | 0.89 |
|  |  |  |  | IN-ND – IV | 0.78 | 0.10 | 1.00 | 1.00 |
|  |  |  |  | IN-ND 50µl - IV | 0.85 | 0.71 | 0.49 | 0.47 |
|  |  |  |  | IN-NTB – SC | 0.99 | 0.98 | 0.67 | 0.82 |
|  |  |  |  | **IN-ND** - SC | 0.064 | 0.042 | 0.99 | 0.99 |
|  |  |  |  | IN-ND 50µl - SC | 0.94 | 0.67 | 0.28 | 0.29 |
|  |  |  | 20 | **IN-NTB** - **IV** | <0.001 (IV) | 0.73 | 0.001 (IN) | <0.001 (IN) |
|  |  |  |  | **IN-ND** **– IV** | 0.014 (IV) | 0.044 (IN) | 0.93 | 0.87 |
|  |  |  |  | IN-ND 50µl - **IV** | <0.001 | 0.99 | 0.61 | 0.49 |
|  |  |  |  | **IN-NTB** – SC | 0.72 | 0.090 | <0.001 | <0.001 |
|  |  |  |  | **IN-ND** - SC | 0.040 | <0.001 | 0.77 | 0.76 |
|  |  |  |  | IN-ND 50µl - SC | 0.50 | 0.46 | 0.31 | 0.29 |
| JNJ-01 | 20% PEG | 5 | 5 | IN-NTB - **IV** | <0.001 | 0.0037 | <0.001 | 0.40 |
|  |  |  | 20 | IN-NTB - **IV** | <0.001 | <0.001 | <0.001 | <0.001 |
|  | 40% SBEbCD | 5 | 5 | IN-NTB - **IV** | 0.0033 | 0.29 | 0.0012 | 0.26 |
|  |  |  | 20 | IN-NTB **- IV** | <0.001 | 0.0012 | <0.001 | 0.0013 |
|  | McIlvain buffer | 5 | 5 | IN-NTB - **IV** | <0.001 | <0.001 | <0.001 | <0.001 |
|  |  |  | 20 | IN-NTB - **IV** | <0.001 | <0.001 | <0.001 | 0.0021 |
| JNJ-02 | 40% SBEbCD | 3 | 5 | IN-NTB - IV | 0.42 | 0.37 | 0.26 | 0.25 |
| JNJ-03 | 20% SBEbCD | 1.2 | 5 | **IN-NTB** - IV | 0.12 | 0.039 | 0.13 | 0.14 |
|  | 40% PEG400 | 0.25 | 5 | **IN-NTB** - IV | 0.20 | 0.015 | <0.001 | <0.001 |
|  | 40% SBEbCD | 0.25 | 5 | IN-NTB - IV | 0.10 | 0.070 | 0.067 | 0.053 |
|  |  | 1.2 | 5 | **IN-NTB** - IV | 0.16 | 0.11 | 0.053 | 0.016 |
|  |  | 3 | 5 | **IN-NTB** - IV | 0.0044 | 0.0029 | 0.0018 | 0.0014 |
| JNJ-04 | 0,9% NaCl | 3 | 5 | IN-NTB - IV | 0.48 | 0.60 | 0.22 | 0.28 |
|  | 40% SBEbCD | 3 | 5 | **IN-NTB** - IV | <0.001 | <0.001 | <0.001 | <0.001 |
| JNJ-05 | 40% SBEbCD | 2.4 | 30 | IN-NTB - IV | 0.67 | 0.79 | 0.25 | 0.22 |
|  |  |  | 60 | **IN-NTB** - IV | - | - | 0.013 | 0.004 |
|  |  |  | 180 | **IN-NTB** - IV | 0.13 | 0.12 | <0.001 | <0.001 |
|  |  |  | 360 | **IN-NTB** - IV | 0.23 | 0.22 | 0.0078 | 0.011 |
| JNJ-06 | 40% SBEbCD | 1.2 | 5 | **IN-NTB** - IV | 0.15 | <0.001 | 0.052 | 0.033 |
