# Supplementary material for: Direct nose to brain delivery of small molecules: critical analysis of data from a standardized in vivo screening model in rats
Source: Drug Deliv. 2020 Nov 10;27(1):1597–607. doi: 10.1080/10717544.2020.1837291 (PMC7655051; doi:10.1080/10717544.2020.1837291)

**Supplementary data**

7° Distribution of [^3^H]-Morphine in rat brain 5 min after IN-ND dosing (16 µCi/rat). The figure illustrates autoradiography images of left and right hemisphere of 5 individual animals (A1-5), including hematoxylin counterstain on the same sections. The thin arrow indicates the olfactory bulb, the wide arrow indicates the brain stem.


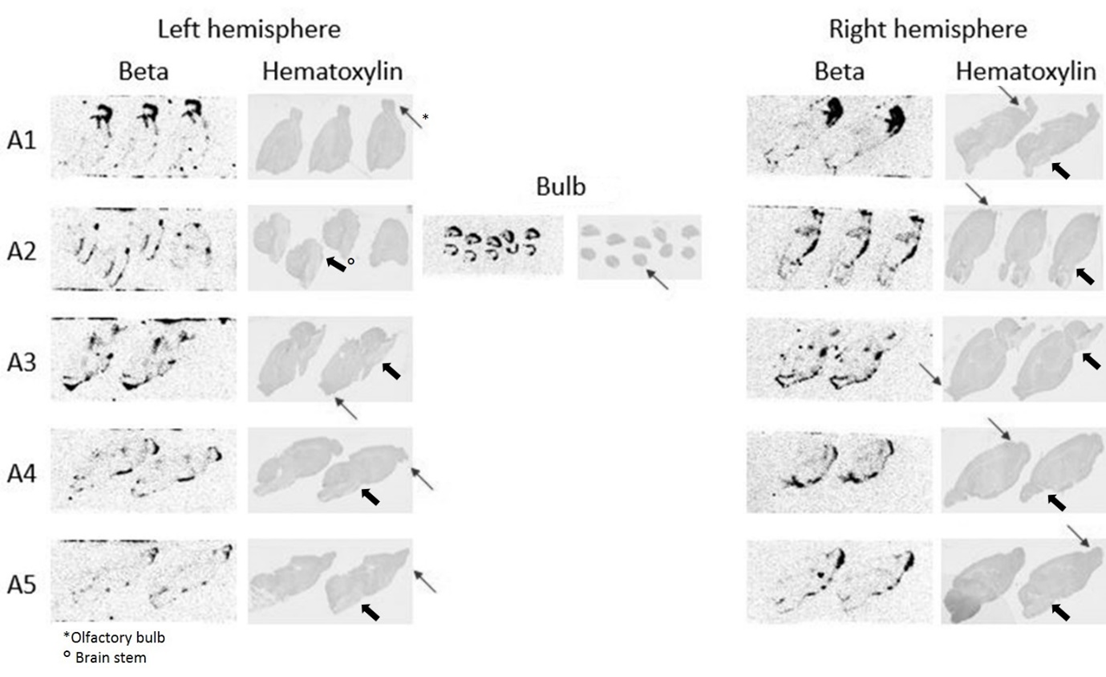

Supplement: Supplemental Material [file IDRD_A_1837291_SM6603.zip › Manuscript_NTB_Dhuyvetter_Suppl7.docx]
